# 2308. Exploring the Seasonality of COVID-19 with Machine Learning: A Distinct Bimodal Pattern?

**DOI:** 10.1093/ofid/ofad500.1930

**Published:** 2023-11-27

**Authors:** Guillermo Rodriguez-Nava, Lucy S Tompkins, Karen McIntyre, Bryan Bohman, Jorge Salinas

**Affiliations:** Stanford University School of Medicine, Palo Alto, California; Stanford University, PORTOLA VALLEY, California; Stanford Healthcare, Stanford, California; Stanford School of Medicine, Emerald Hills, California; Stanford University, PORTOLA VALLEY, California

## Abstract

**Background:**

Respiratory viruses, such as influenza and RSV, occur annually during the winter season in temperate regions. It is essential to determine if SARS-CoV-2 exhibits seasonal patterns for public health planning and vaccination policies. Although winter spikes in COVID-19 cases have been observed, the higher transmissibility of SARS-CoV-2 suggests its seasonality may have a different pattern.

**Methods:**

We plotted the time-series data on COVID-19, Influenza, and RSV from January 2020-February 2023 at Stanford Healthcare, to compare seasonal patterns. We then modeled predictions for COVID-19 cases in the United States for the next 913 days, using data from January 2020-February 2023. This data was split into a training set and a test set, then Facebook Prophet machine learning library was used to fit a model to the training set and generate forecasts for the test set and future time periods. Cross-validation of the model was performed by calculating the root mean squared error (RMSE) and mean absolute percentage error (MAPE).

**Results:**

The time-series data of cases for Influenza, RSV, and COVID-19 were plotted from January 1, 2020, to February 28, 2023, revealing bimodal peaks for COVID-19 during the winter (December - February) and summer (July - September) months **(Figure 1)**. This differed from the unimodal patterns observed for Influenza and RSV cases **(Figures 2 and 3, respectively).** The Prophet model for COVID-19 cases over the next 913 days in the United States demonstrated an additional peak during the summer months **(Figure 4**). However, other external factors that may influence seasonality, such as the stochastic development of new SARS-CoV-2 variants, weaning of immunity, and testing intensity were not modeled.

**Figure 1.**
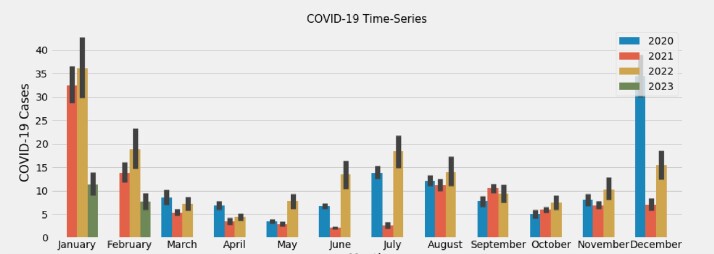
COVID-19 time-series at Stanford Healthcare during January 2020–February 2023 Time-series of COVID-19 cases demonstrates bimodal peaks during the winter (December - February) and summer (July - September) months.

**Figure 2.**
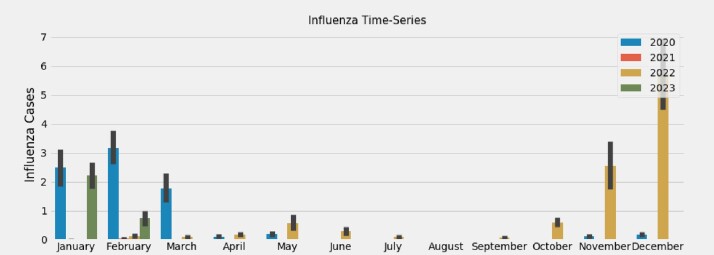
Influenza time-series at Stanford Healthcare during January 2020–February 2023 Time-series of Influenza cases demonstrates one peak during the winter (December - February) months.

**Figure 3.**
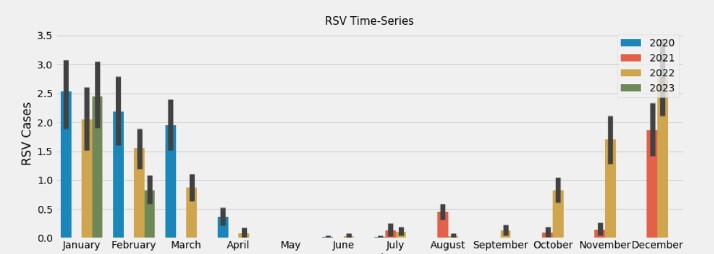
RSV time-series at Stanford Healthcare during January 2020–February 2023 Time-series of RSV cases demonstrates one peak during the winter (December - February) months.

**Conclusion:**

COVID-19 may exhibit distinct bimodal seasonal patterns that are not observed for other respiratory viruses. Future public health interventions should consider the distinct seasonal patterns of COVID-19 when developing prevention and control strategies.

**Figure 4.**
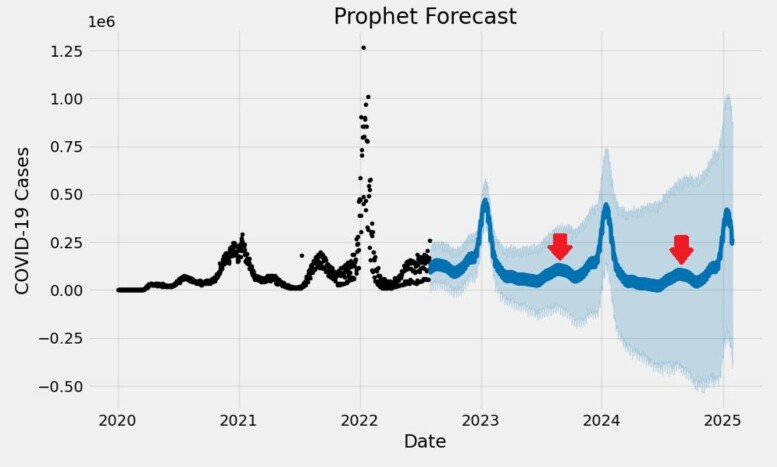
Prophet Model forecasting COVID-19 peaks in the United States for the next 3 years

Cross-validation results for the Prophet model's performance in predicting COVID-19 cases in the United States over a 5-day horizon. The model's RMSE was 110,342.81, indicating an average deviation from the actual values. The MAPE was 0.486, reflecting the model's average percentage error. The coverage was 0.35, indicating that the actual value fell within the 80% prediction interval of the model only 35% of the time, which may suggest limitations for longer-term forecasting.

**Disclosures:**

**All Authors**: No reported disclosures

